# TTC7B is a new prognostic biomarker in head and neck squamous cell carcinoma linked to immune infiltration and ferroptosis

**DOI:** 10.1002/cam4.6715

**Published:** 2023-11-22

**Authors:** Rong He, Xun Zhang, Yongzhi Wu, Zhijie Weng, Longjiang Li

**Affiliations:** ^1^ State Key Laboratory of Oral Diseases, National Clinical Research Center for Oral Diseases, Department of Head and Neck Oncology, West China Hospital of Stomatology Sichuan University Chengdu China; ^2^ Guangyuan Hospital of Traditional Chinese Medicine Guangyuan China

**Keywords:** head and neck squamous cell carcinoma, focal adhesions, immune infiltration, ferroptosis, TTC7B

## Abstract

**Objective:**

To investigate the expression of TTC7B and its prognostic significance, biological roles, and impact on the immune system in patients with head and neck squamous cell carcinoma (HNSCC).

**Materials and Methods:**

Clinical and genomic data were obtained from TCGA (The Cancer Genome Atlas), GEO (Gene Expression Omnibus), GEPIA2 (Gene Expression Profiling Interactive Analysis 2.0), and TIMER2.0 (Tumor Immune Estimation Resource 2.0) databases. R software was utilized to process the retrieved data. qPCR and immunohistochemical assays were performed to validate the findings obtained from the databases.

**Results:**

High expression of TTC7B was observed in HNSCC, and this heightened expression is significantly associated with reduced overall survival (OS) in patients, making it an independent risk factor impacting OS. TTC7B is correlated with focal adhesions and cell migration pathways based on functional enrichment analysis. CIBERSORT analysis and TIMER2.0 show a positive link between TTC7B and multiple immune cells, particularly macrophages. Pearson's analysis reveals a significant correlation between TTC7B and ferroptosis‐related genes.

**Conclusion:**

In all, TTC7B could serve as a promising prognostic indicator of HNSCC, and is closely associated with focal adhesions, immune infiltration, and ferroptosis.

## 1 INTRODUCTION

Head and neck squamous cell carcinoma (HNSCC), which develop from the mucosal epithelium in the oral cavity, pharynx, and larynx, is the sixth most common cancer worldwide (890,000 new cases and 450,000 deaths annually), contributing to the rapidly increasing burden of cancer incidence and mortality.^1^ HNSCC exhibit remarkable heterogeneity, and despite significant advances in diagnosis and treatment that have improved the prognosis for patients, the challenges of metastasis and recurrence persist.^2^ The physical pain and financial burden endured by HNSCC patients make it imperative to identify optimal diagnostic and therapeutic targets to enhance their outcomes and alleviate their suffering.

Tetratricopeptide repeat domain 7B (TTC7B), a member of the tetratricopeptide repeat (TPR) gene family, is a protein‐coding gene linked to several diseases, including ischemic stroke and glioma.^3^, ^4^ While TTC7B has not been previously reported in HNSCC patients to date, there have been numerous explorations of hot spots in cancer therapy, such as tumor immunotherapy and ferroptosis, in various HNSCC‐related studies and treatments.^5^, ^6^, ^7^ In this study, we aim to analyze the role of TTC7B in HNSCC from multiple perspectives, including functional enrichment, immune infiltration, and ferroptosis. By delving into these aspects, we hope to shed light on the potential significance of TTC7B in the context of HNSCC and its possible implications for novel therapeutic approaches.

## 2 MATERIALS AND METHODS

### 2.1 Downloading and preprocessing of HNSCC datasets

Clinical and transcriptome data of patients with HNSCC were acquired from two reputable databases, the TCGA (http://cancergenome.nih.gov/) and GEO (https://www.ncbi.nlm.nih.gov/geo/) database. From the TCGA database, 520 tumor specimens and 44 normal specimens were obtained. In addition, we obtained specimens from GEO database (GSE184616, GSE3292, GSE65858, GSE74927, GSE41613) to verify the result of the analysis with TCGA database. The clinicopathological characteristics of HNSCC patients, such as age, gender, overall survival (OS), censor and TNM stage of American Joint Committee on Cancer (AJCC), were extracted from TCGA database (*n* = 520).^8^ Data preprocessing was performed on the different datasets, and various correlation analyses were conducted using R software (version 4.3.0).

### 2.2 Investigation of expression and survival

Initially, we utilized the TIMER2.0 online database to conduct an analysis of TTC7B expression across various tumor types. Subsequently, the GEPIA2 (Gene Expression Profiling Interactive Analysis 2, http://gepia2.cancer‐pku.cn/index.html) online repository was employed to investigate the differential expression of TTC7B between HNSCC and normal tissues. Furthermore, we assessed the correlation between TTC7B expression and the survival of patients with HNSCC using data from the GEPIA2 website. The GEPIA2 platform offers a comprehensive RNA sequence expression profile encompassing 198,619 isoforms and 84 cancer subtypes derived from the TCGA and GTEx samples.^9^ To further corroborate the results obtained from GEPIA2, we conducted additional analyses using the TCGA‐HNSCC and GEO datasets with IBM SPSS Statistics 26 software (SPSS) and GraphPad Prism 9 software. We again proceeded to classify the samples within TCGA‐HNSCC based on their origins in the oral cavity, pharynx, and larynx. However, due to the ambiguous classification of samples from the oral cavity and pharynx within TCGA‐HNSCC, we opted to conduct an analysis grouping these two regions together. To ensure the robustness of our results, we performed cross‐validation by comparing our TCGA‐HNSCC dataset findings with additional data from the GEO database.

### 2.3 Univariate and multivariate analyses of prognostic parameters

Univariate and multivariate analyses of prognostic parameters were performed to assess the correlation between age, gender, smoking history, clinical stage of AJCC, and TTC7B expression with OS in the TCGA‐HNSCC datasets. For conducting the aforementioned analyses, we utilized SPSS software, ensuring a rigorous and comprehensive evaluation of the data.

### 2.4 Functional enrichment analysis

The most relevant genes of TTC7B derived from TCGA‐HNSCC and GSE184616 datasets were submitted to The Database for Annotation, Visualization and Integrated Discovery (DAVID,https://david.ncifcrf.gov/). As an identity, the official gene symbol was chosen, and Homo sapiens was selected as the species. The results of the Kyoto Encyclopedia of Genes and Genomes (KEGG) pathway analysis enrichment as well as Gene Ontology (GO) analysis were obtained.^10^ In this study, we present the top six results in ascending order of *p*‐value (*p* < 0.05), thereby highlighting the most significant enriched pathways and functions associated with TTC7B in the context of HNSCC.^11^

### 2.5 Gene set variation analysis (GSVA)

To analyze the gene sets associated with specific bioprocesses and pathways, we obtained the gene list from GeneSets (http://baderlab.org/GeneSets). The functional enrichment score for each HNSCC sample was calculated using the provided R software with default parameters. The R package “pheatmap” was utilized to visualize the enrichment results. Pearson correlation analysis was conducted to determine the relationship between TTC7B and the selected bioprocesses and pathways.^11^

### 2.6 CIBERSORT analysis

CIBERSORT is a computational method for characterizing cell composition of complex tissues based on gene expression profiles.^12^ LM22, which is an annotated gene profile matrix representing the 22 distinct kinds of immune cells. In the TCGA‐HNSCC and GSE184616 datasets, based on the “CIBERSORT” R, the LM22 gene signature matrix was used in to assess the proportion of immune cells in HNSCC.^13^ The calculated data were categorized into high and low TTC7B expression groups based on the median expression levels of TTC7B. The proportion of immune cells in HNSCC samples with high and low TTC7B expression were examined using the R software package. The final results were visualized using the “ggboxplot” package in R.

### 2.7 TIMER 2.0 database analysis

TIMER2.0 (https://cistrome.shinyapps.io/timer/), an updated version of TIMER, is a web server that allows comprehensive analysis of tumor‐infiltrating immune cells in over 30 cancer types.^14^ In this study, we utilized TIMER2.0 to evaluate the relationship between TTC7B and immune cells, including B cells, CD4^+^ T cells, CD8^+^ T cells, natural killer cell, macrophages, dendritic cells, and neutrophils in HNSCC. We also analyze the somatic copy number alterations (SCNA) in TTC7B and investigated the relationship between immune cell levels and cumulative survival in HNSCC.

### 2.8 Correlations of TTC7B expression with ferroptosis‐related genes

The relationship between TTC7B expression and ferroptosis‐related genes was analyzed in the TCGA‐HNSCC and GSE184616 datasets using the R software.^5^ The fraction of ferroptosis‐related genes in HNSCC samples with high and low TTC7B expression was examined using the R software package. The final results were visualized using the “ggboxplot” function in R.^15^

### 2.9 Quantitative reverse transcription polymerase chain reaction (qRT–PCR)

The head and neck squamous carcinoma (HNSCC) cell lines CAL‐27, SCC‐25, HSC‐3, and human oral keratinocytes line HOK were acquired from the State Key Laboratory of Oral Diseases, Chengdu, China. HNSCC and adjacent paracancerous tissue samples were procured from West China Stomatological Hospital in Sichuan, China. Total RNA was extracted using the Trizol‐based method according to the manufacturer's instructions (Servicebio). RevertAid First Strand Cdna Synthesis Kit was used for the reverse transcription of the total RNA. (Thermo Scientific, RevertAid First Strand Cdna Synthesis Kit). 2× SYBR Green Qpcr Master Mix (Low ROX) was used for qRT‐PCR according to the protocol (Bimake.com). The relative gene expression of TTC7B was calculated by the method of 2^−ΔΔCt^ with GAPDH gene expression as a control. All primers were synthesized by Sangon Biotech. The primer sequences used were as follows: GAPDH forward, 5′‐GGAGCGAGATCCCTCCAAAAT‐3′ and reverse, 5′‐GGCTGTTGTCATACTTCTCATGG‐3′; TTC7B forward, 5′‐GCAGAAGCCTACGCTACCAAA‐3′ and reverse, 5′‐AGATACAGGAGTGCGATGTCC‐3′.

### 2.10 Immunohistochemistry

Formalin‐fixed paraffin‐embedded tissue blocks of HNSCC were obtained from West China Stomatological Hospital, Sichuan, China, and then sectioned into 4‐μm‐thick slices using a microtome and mounted onto glass slides. The tissue sections were deparaffinized in xylene and rehydrated through a series of graded ethanol solutions. Heat‐induced antigen retrieval was performed by microwaving the slides in citrate buffer. Nonspecific binding was blocked using 3% hydrogen peroxide and normal serum. The tissue sections were incubated with the primary antibody of TTC7B (A12170‐1, BOSTER, Wuhan, China) overnight at 4°C. After washing, the tissue sections were incubated with the corresponding biotinylated secondary antibody (ab6721, abcam, Shanghai, China). Streptavidin‐HRP conjugate was applied, and the antigen–antibody complex was visualized using DAB as the chromogen. The tissue sections were counterstained with hematoxylin, dehydrated, and mounted with coverslips. Images of the immunostained tissue sections were scanned and digitized using a KF‐PRO‐005‐EX scanner (KFBIO, Ningbo, China) at a magnification of 20‐fold and saved in KFB format.

## 3 STATISTICAL ANALYSES

Experiments, comprising qRT‐PCR and immunohistochemistry were conducted in triplicate to ensure robustness and accuracy. Data are expressed as the mean ± SD. To compare two groups of data, an unpaired Student's *t*‐test was employed. Statistical significance was established as *p* < 0.05, indicating the reliability and significance of the observed results.

## 4 RESULTS

### 4.1 Pan‐Cancer analysis of TTC7B mRNA expression in TIMER2.0

**FIGURE 1:**
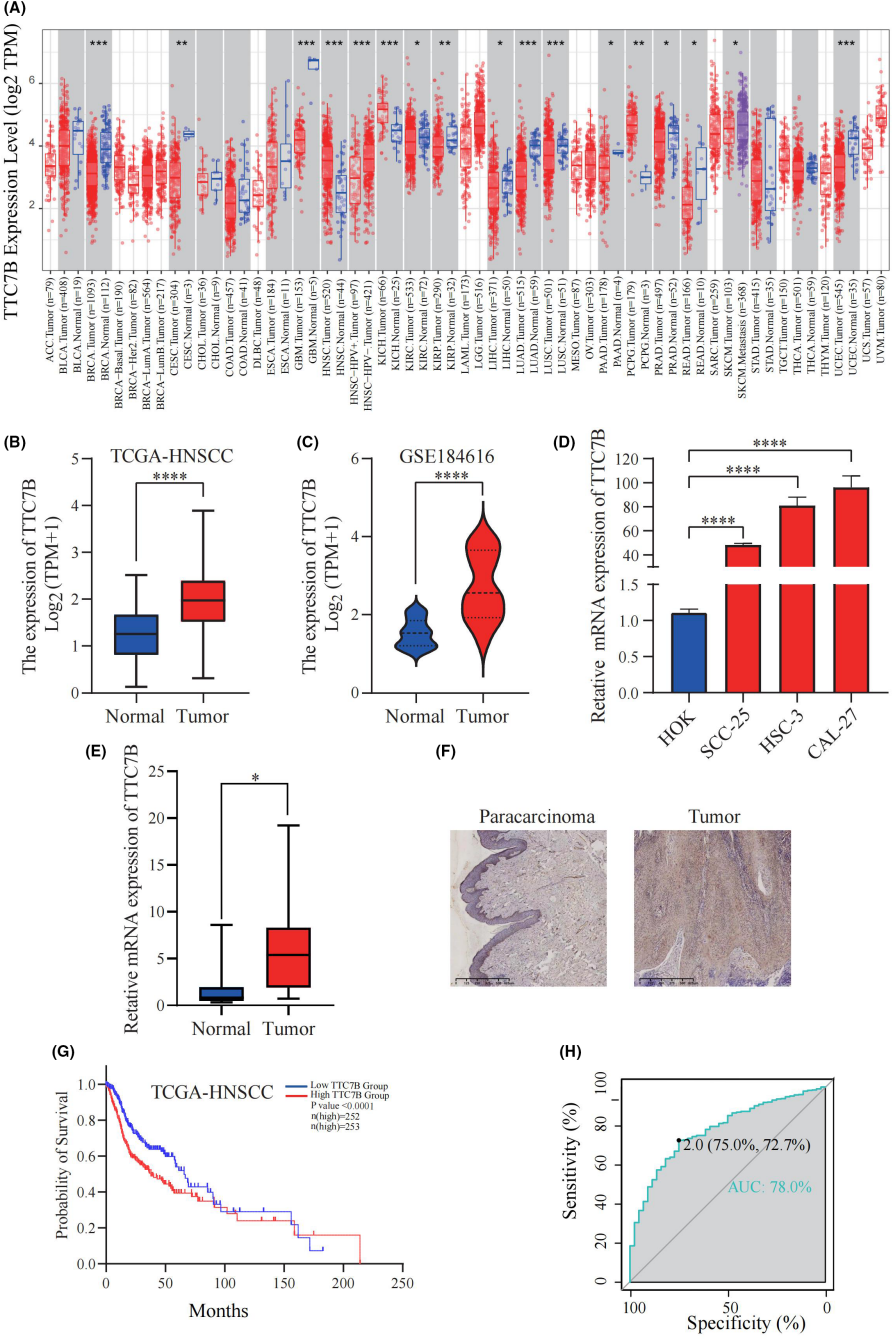
The expression of TTC7B in Head and neck squamous cell carcinoma (HNSCC) and pan‐carcinoma. (A) TTC7B mRNA expression levels in pan‐cancer were measured using TIMER2.0. (B) Difference in expression of TTC7B between HNSCC and matched normal tissues in The Cancer Genome Atlas (TCGA) database. (C) Difference in TTC7B expression between HNSCC and normal tissues in GSE184616 datasets. (D) Difference in TTC7B expression between HNSCC cell lines and human oral mucosal keratinocytes cell lines. (E) Difference in TTC7B expression between HNSCC tissues and paracarcinoma tissues. (F) Immunohistochemistry assay was used to analyze the expression of TTC7B in HNSCC tissues and paracarcinoma tissues. (G) The survival curve of TTC7B in TCGA database. (H) The receiver‐operating characteristic (ROC) curve showed the high‐expression specificity of TTC7B in HNSCC diagnosis. *p < 0.05; **p < 0.01; ***p < 0.001; ****p < 0.0001. NS, not significant.

The analysis from the TIMER2.0 database revealed that the expression of TTC7B in breast invasive carcinoma, cervical squamous cell carcinoma and endocervical adenocarcinoma, glioblastoma multiforme, kidney renal clear cell carcinoma, kidney renal papillary cell carcinoma, liver hepatocellular carcinoma, lung adenocarcinoma, lung squamous cell carcinoma, pancreatic adenocarcinoma, prostate adenocarcinoma, rectum adenocarcinoma, and uterine corpus endometrial carcinoma was lower than that in normal tissues. However, in head and neck squamous cell carcinoma, kidney chromophobe, and pheochromocytoma & paraganglioma, the expression of TTC7B was higher than that in normal tissues. Additionally, we observed that the expression of TTC7B in HPV‐negative HNSCC was higher than that in HPV‐positive HNSCC (Figure 1A).

### 4.2 Expression levels of TTC7B in HNSCC patients

We conducted a detailed analysis of the differential expression of TTC7B between HNSCC and normal tissues in both TCGA‐HNSCC and GEO datasets (Figure 1B,C). The results obtained were consistent with those from TIMER2.0 analysis, reinforcing the validity of our findings. Further categorizing HNSCC by anatomical site led to comparable findings (Figure S1a,b). Considering that HPV‐positive HNSCC typically exhibits a more favorable prognosis than HPV‐negative HNSCC, we leveraged various databases to investigate whether there exists a discrepancy in TTC7B expression between these two subtypes. Our findings revealed that TTC7B expression was notably higher in HPV‐negative HNSCC when compared to HPV‐positive HNSCC (Figure S1c,d). Moreover, our qPCR results demonstrated a significant upregulation of TTC7B in HNSCC cell lines compared to HOK cells (Figure 1D). Similarly, the qPCR and immunohistochemistry experiments showed a marked increase in TTC7B expression in HNSCC tissues compared to paracancerous tissues (Figure 1E,F).

### 4.3 Relationship between TTC7B expression and HNSCC survival rates

HNSCC samples were categorized into high and low TTC7B expression groups based on the median TTC7B expression levels. Notably, GEPIA2 analysis demonstrated that high TTC7B expression was associated with unfavorable OS (*p* < 0.001) (Figure S1f). Additionally, the Kaplan–Meier curve derived from the TCGA‐HNSCC dataset exhibited a similar trend, reinforcing the significance of the findings and corroborating the results obtained from GEPIA2 analysis (*p* < 0.05) (Figure 1G).

### 4.4 TTC7B is an independent predictive indicator for HNSCC patients

In the univariate analysis, age (*p* < 0.05), gender (*p* < 0.05), clinical metastasis (*p* < 0.05), and TTC7B expression (*p* < 0.001) were identified as factors associated with the prognosis of HNSCC patients. However, in the multivariate analysis, only age (*p* < 0.05) and TTC7B expression (*p* < 0.001) emerged as independent predictive indicators for HNSCC patients (Table 1). Stratifying based on anatomical site revealed that elevated TTC7B expression in HNSCC‐oral cavity & pharynx correlated with an unfavorable prognosis, while no significant difference was observed in HNSCC‐larynx (Table S1). The receiver‐operating characteristic (ROC) analysis utilizing TTC7B transcriptional data from tumor and control samples in the TCGA database revealed an area under the ROC curve (AUC) of 0.780. Additionally, TTC7B demonstrated a sensitivity of 0.750 and specificity of 0.727 as a diagnostic biomarker for HNSCC (Figure 1H). These results signify that TTC7B holds promise as a potential biomarker for diagnosing HNSCC.

**TABLE 1:** Univariate and multivariate analyses of prognostic parameters in The Cancer Genome Atlas (TCGA) database.

Parameter	Univariate analysis			Multivariate analysis		
	HR	95% CI	*p*	HR	95% CI	*p*
Age	1.021	1.008–1.033	0.001	1.019	1.006–1.032	0.004
Gender	1.391	1.046–1.850	0.023	1.122	0.969–1.301	0.124
Smoking history	0.978	0.854–1.120	0.749			
Clinical_M	0.357	0.132–0.962	0.042	0.648	0.394–1.066	0.088
Clinical_N	0.827	0.634–1.079	0.161			
Clinical_T	0.775	0.582–1.032	0.081			
Clinical_stage	0.841	0.608–1.162	0.293			
TTC7B	1.424	1.165–1.741	0.000	1.433	1.176–1.759	0.000

### 4.5 Analysis of TTC7B‐related functions using DAVID

**FIGURE 2:**
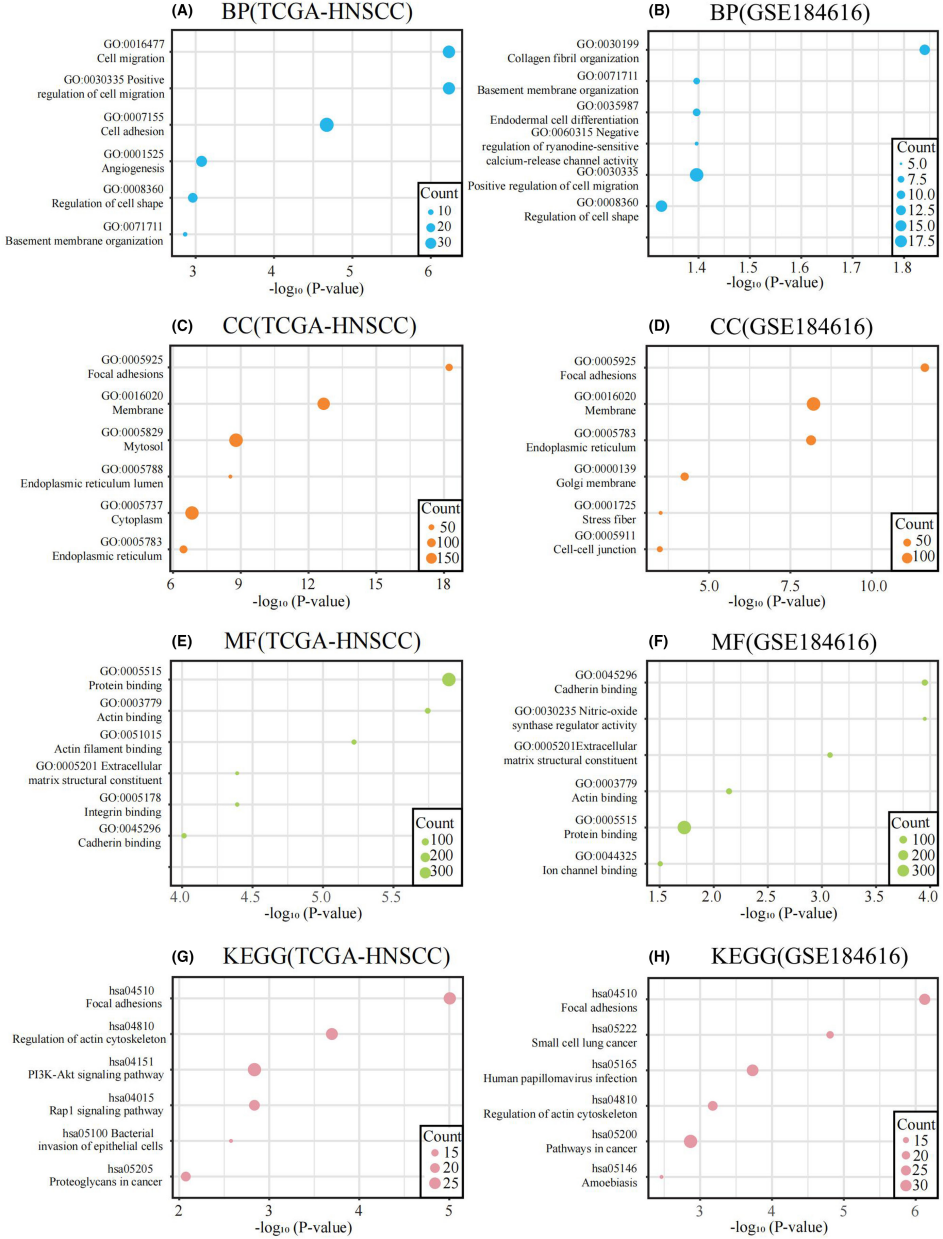
TTC7B is closely associated with focal adhesion, angiogenesis and cell migration in Head and neck squamous cell carcinoma (HNSCC). (A, C, E) Biological processes (BP), cellular components (CC), and molecular functions (MF) are mostly related to TTC7B in The Cancer Genome Atlas (TCGA) database. (G) Kyoto Encyclopedia of Genes and Genomes (KEGG) pathway analysis of TTC7B in the TCGA database. (B, D, F) Biological processes (BP), cellular components (CC), and molecular functions (MF) are mostly related to TTC7B in the GSE184616 datasets. (H) Kyoto Encyclopedia of Genes and Genomes (KEGG) pathway analysis of TTC7B in the GSE184616 datasets.

To unravel the biological functions associated with TTC7B, we conducted Pearson correlation analysis to identify genes most closely linked to TTC7B in the TCGA‐HNSCC and GSE184616 datasets. Subsequently, we selected the top 500 genes with the highest R‐values and a significance level of *p* < 0.05 for further functional analysis using the DAVID website. The Gene Ontology (GO) analysis revealed significant biological processes (BP) associated with TTC7B, including positive regulation of cell migration, regulation of cell shape, and basement membrane organization (Figure 2A, B). Furthermore, TTC7B was found to be involved in crucial cellular components (CC) such as focal adhesions (FAs), membrane, and endoplasmic reticulum (Figure 2C, D). Regarding molecular functions (MF), TTC7B exhibited connections to protein binding, actin binding, extracellular matrix structural constituent, and cadherin binding (Figure 2E, F). Additionally, the most relevant signaling pathways associated with TTC7B were found to be FAs and regulation of the actin cytoskeleton (Figure 2G, H). Importantly, FAs and cell migration are known to play vital roles in tumor progression. These findings suggest that TTC7B may significantly contribute to tumor progression by participating in multiple important cellular functions.

### 4.6 Positive correlation between TTC7B expression and FAs in HNSCC cells

**FIGURE 3:**
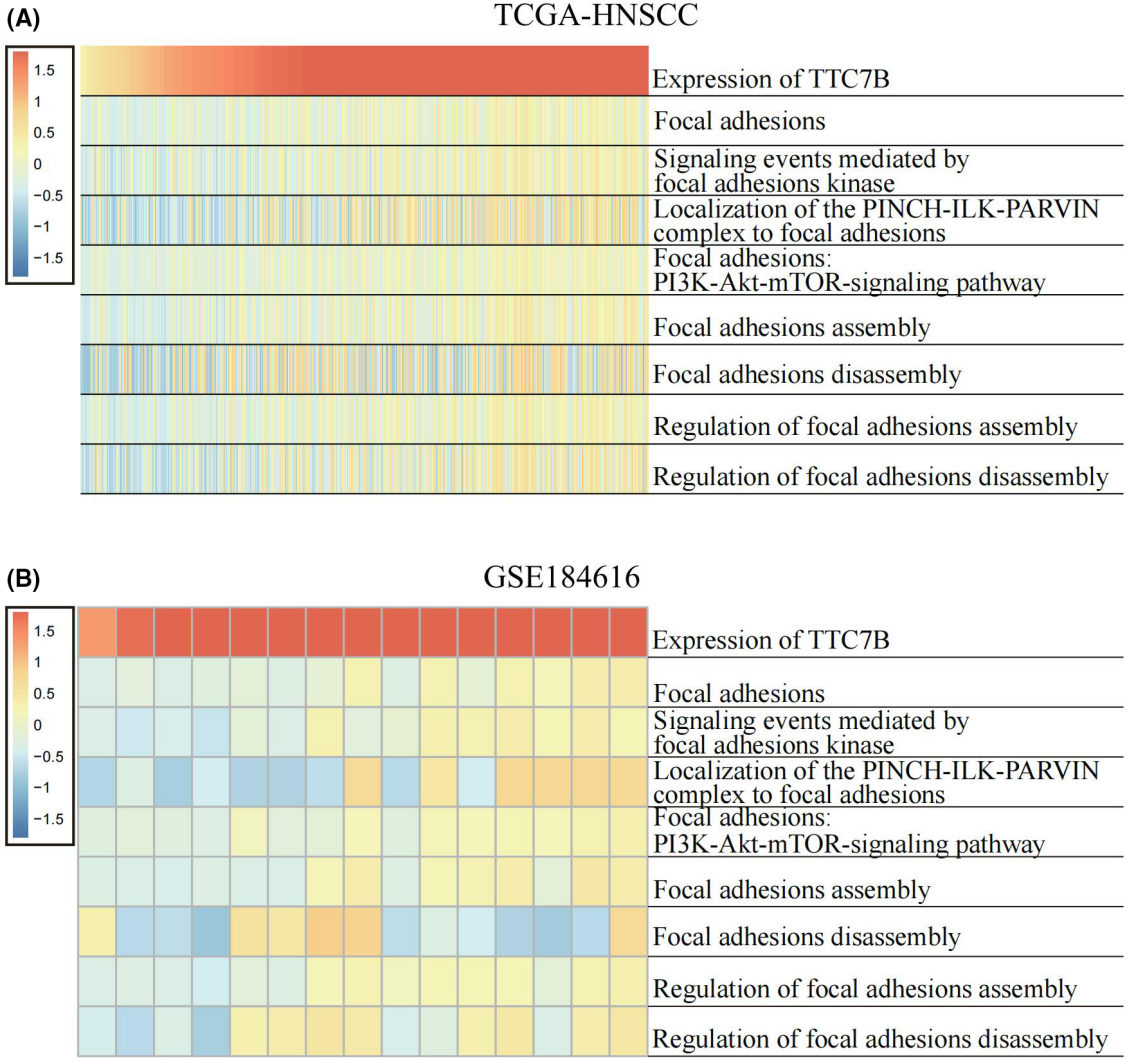
Correlation analysis between TTC7B expression and focal adhesion‐related gene lists enrichment scores in Head and neck squamous cell carcinoma (HNSCC). (A, B) The heatmap showed the expression of TTC7B and the enrichment scores of focal adhesion‐related gene lists of each patient in The Cancer Genome Atlas (TCGA) and the GSE184616 datasets. The samples were arranged in ascending order of the expression of TTC7B.

Given the importance of FAs in angiogenesis and cell migration, we further investigated the relationship between TTC7B and FAs. FAs are the most well characterized integrin adhesion complexes (IACs). Acting like anchoring units and bidirectional link between the cell and its extra cellular matrix (ECM), FAs are essential for various functions, especially cell micromovement and migration.^16^ As FAs‐related proteins have been known to be elevated in various cancers, we conducted gene set variation analysis (GSVA) in the TCGA‐HNSCC and GSE184616 datasets to explore the connection between FAs and TTC7B expression in HNSCC.^16^, ^17^, ^18^ The analysis revealed a positive correlation between TTC7B expression and the enrichment scores of all pathways involved in FAs (Figure 3). Specifically, the enrichment scores of “localization of the PINCH‐ILK‐PARVIN complex to FAs,” “FAs disassembly,” and “regulation of FAs disassembly” exhibited a stronger correlation with TTC7B expression. These findings indicate a positive association between TTC7B and FAs in HNSCC, particularly with respect to the PINCH‐ILK‐PARVIN complex and FAs disassembly.

### 4.7 Correlation between TTC7B and tumor immune infiltrating cells in HNSCC

**FIGURE 4:**
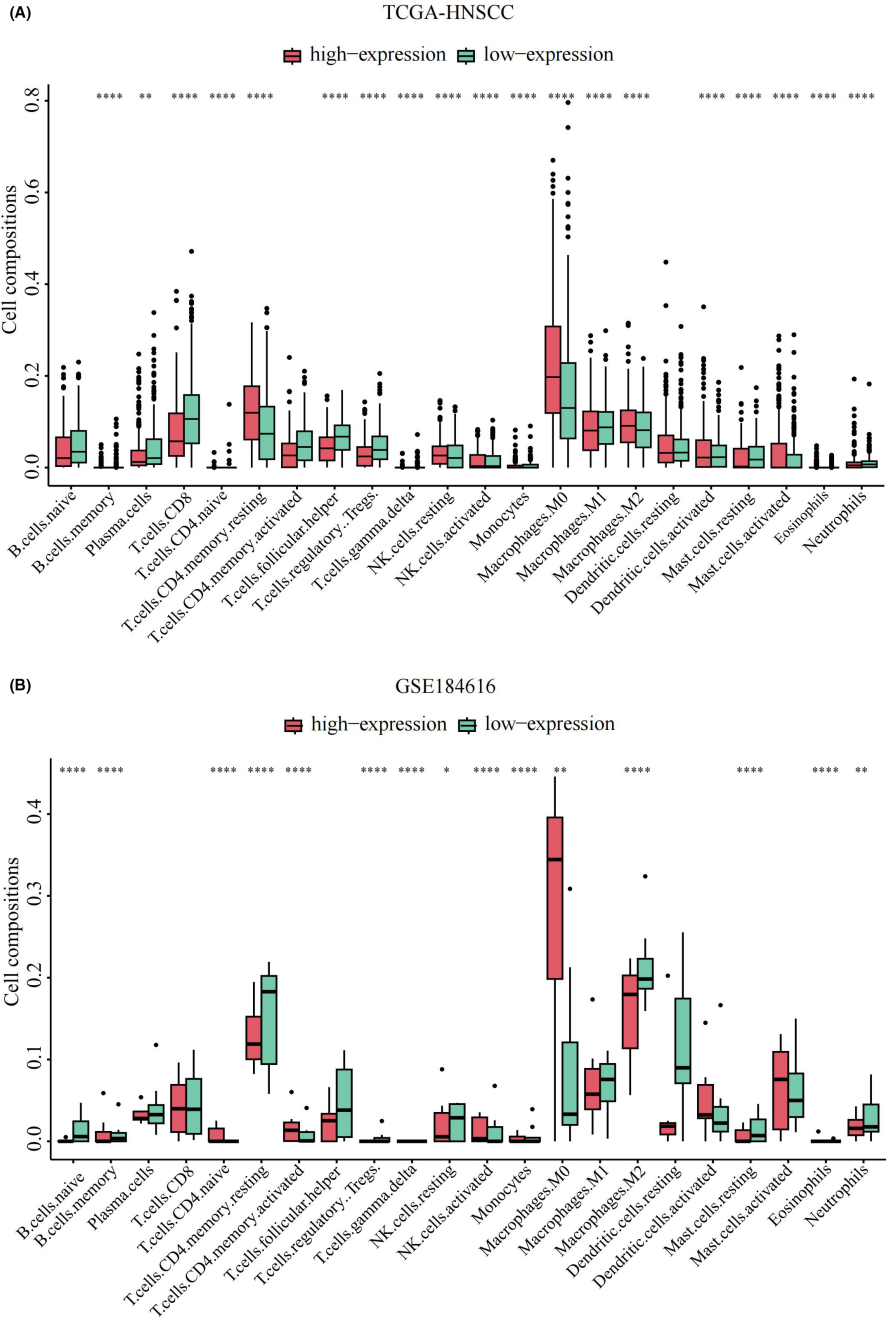
Correlation between TTC7B and tumor immune infiltrating cells in head and neck squamous cell carcinoma (HNSCC) based on CIBRESORT analysis. (A, B) Changes in 22 immune cell subtypes between high and low TTC7B expression groups in The Cancer Genome Atlas (TCGA) and the GSE184616 datasets. *p < 0.05; **p < 0.01; ***p < 0.001; ****p < 0.0001. NS, not significant.

**FIGURE 5:**
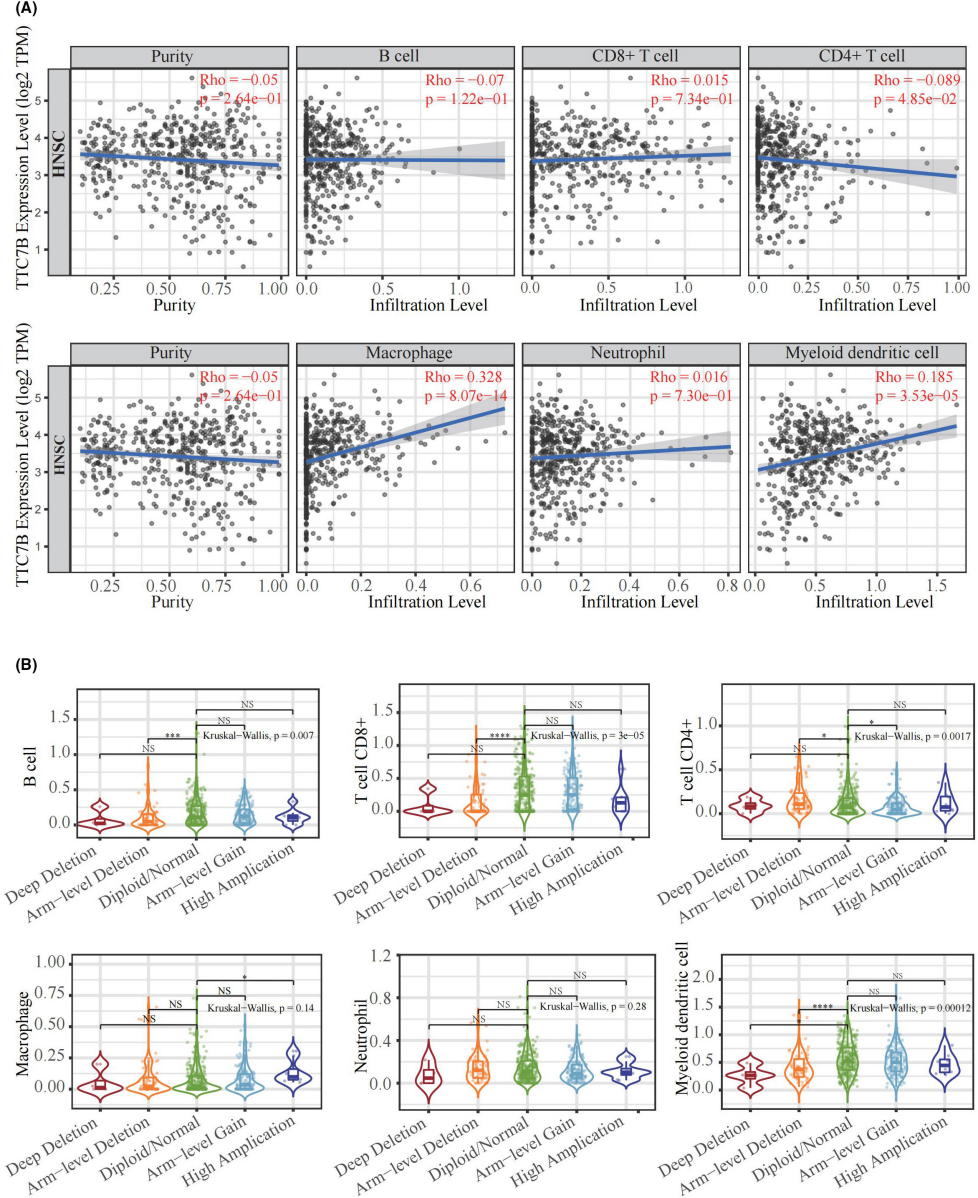
Correlation between TTC7B and tumor immune infiltrating cells in Head and neck squamous cell carcinoma (HNSCC) based on TIMER2.0 online database. (A) Correlation between the expression of TTC7B and immune infiltrating cells in HNSCC. (B) TTC7B somatic copy number alterations (SCNA) affect the infiltrating levels of B cell, CD8^+^ T cell, CD4^+^ T cell, macrophages, and myeloid dendritic cell in HNSCC. *p < 0.05; **p < 0.01; ***p < 0.001; ****p < 0.0001. NS, not significant.

We conducted CIBERSORT analysis using the TCGA‐HNSCC and GSE184616 datasets to examine the correlation between TTC7B expression and tumor immune cell infiltration in HNSCC. The analysis revealed significant correlations between TTC7B expression and the infiltration levels of various immune cells, including B cell memory, T cells CD4 naive, T cells CD4 memory resting, T cells regulatory Tregs, T cells gamma delta, NK cells resting, NK cells activated, monocytes, macrophages M0, macrophages M2, mast cells resting, eosinophils, and neutrophils (Figure 4A, B). Next, we explored the relationship between TTC7B expression and immune infiltrating cells in HNSCC using the TIMER 2.0 database. The results revealed a negative correlation between TTC7B expression and the infiltration levels of B cells (*r* = −0.07, *p* = 1.22e‐01), CD4^+^ T cell (*r* = −0.089, *p* = 4.85e−02) and, while positively correlated with the infiltration levels of macrophages (*r* = 0.328, *p* = 8.07e−14), myeloid dendritic cell (*r* = 0.185, *p* = 3.53e−05), CD8^+^ T cells (*r* = 0.015, *p* = 7.34e−01), neutrophil (*r* = 0.016, *p* = 7.30e−01) (Figure 5A). Notably, we also observed a strong correlation between TTC7B somatic copy number changes (SCNA) and the degree of infiltration by B cells, CD4^+^ T cells, macrophages, neutrophils, and dendritic cells (Figure 5B). These findings suggested the strongest connection between TTC7B expression and macrophage infiltration level.

To further validate the relationship between TTC7B and tumor immune infiltrating cells in HNSCC, we employed TIMER 2.0, GEPIA2 online databases, as well as the TCGA‐HNSCC datasets to explore the association between TTC7B expression and marker genes of several immune cells^15^ (Table 2). The findings from all three databases consistently demonstrated a connection between TTC7B expression and immune cell markers, particularly for macrophages.

**TABLE 2:** Correlation analysis between TTC7B and immune cell marker gene in GEPIA2, TCGA, and TIMER2.0.

Subtype	Gene markers	GEPIA		TCGA		TIMER2.0	
		rho	*p*	rho	*p*	rho	*p*
Tfh	CXCR3	−0.14	**1.10E‐03**	−0.150	**5.92E‐04**	−0.121	**1.38E‐02**
	CXCR5	−0.25	**7.6E−09**	−0.155	**3.90E‐04**	−0.124	**1.71E‐02**
	ICOS	−0.0053	9.00E‐01	−0.019	6.63E‐01	−0.018	8.03E‐01
Th1	CCR1	0.17	**1.00E‐04**	0.177	**4.71E‐05**	0.156	**7.95E‐04**
	CCR5	−0.056	2.00E‐01	−0.05	2.51E‐01	−0.034	6.25E‐01
	IL12RB1	−0.011	8.10E‐01	−0.041	3.56E‐01	−0.024	7.89E‐01
Th2	CCR4	0.033	4.50E‐01	−0.011	8.05E‐01	0.049	3.67E‐01
	CCR8	0.15	**4.10E‐04**	0.142	**1.13E‐03**	0.142	**3.76E‐03**
	HAVCR1	0.011	8.00E‐01	−0.0409	3.52E‐01	−0.046	5.77E‐01
Th17	IL21R	−0.13	**4.20E‐03**	−0.111	**1.13E‐02**	−0.034	6.29E‐01
	IL23R	−0.043	3.30E‐01	−0.074	9.16E‐02	−0.068	2.66E‐01
	CCR6	−0.064	1.50E‐01	−0.151	**5.67E‐04**	−0.009	8.90E‐01
Treg	FOXP3	0.019	6.60E‐01	0.042	3.34E‐01	0.064	1.78E‐01
	NT5E	0.27	**2.3E−10**	0.392	**1.60E‐20**	0.357	**5.25E‐16**
	IL7R	0.29	**2.8E−11**	0.304	**1.41E‐12**	0.319	**8.98E‐13**
M1	CD68	0.31	**2.3E−13**	0.191	**1.21E‐05**	0.273	**9.84E‐10**
	CD80	0.14	**1.40E‐03**	0.168	**1.20E‐04**	0.149	**2.02E‐03**
	CD86	0.14	**1.30E‐03**	0.142	**1.13E‐03**	0.119	**1.31E‐02**
	NOS2	−0.085	5.20E‐02	−0.187	**1.69E‐05**	−0.09	8.40E‐02
	TLR2	0.14	**1.20E‐03**	0.194	**8.19E‐06**	0.144	**2.00E‐03**
	TLR4	0.19	**8.1E−06**	0.201	**3.66E‐06**	0.207	**5.27E‐06**
M2	CD163	0.1	2.10E‐02	0.218	**5.26E‐07**	0.207	**6.48E‐06**
	MSR1	0.2	**5.7E−06**	0.236	**4.92E‐08**	0.217	**1.70E‐06**
	MRC1	0.21	**1.1E−06**	0.32	**7.38E‐14**	0.323	**2.12E‐13**
	CD209	0.18	**5.2E−05**	0.206	**2.30E‐06**	0.218	**1.75E‐06**
Plasmacytoid DC	CLEC4C	0.035	4.20E‐01	0.017	6.96E‐01	0.035	5.90E‐01
	NRP1	0.41	**0**	0.441	**4.19E‐26**	0.393	**5.79E‐20**
	IL3RA	−0.019	6.60E‐01	−0.057	1.94E‐01	−0.037	5.42E‐01
Myeloid cDC1	THBD	0.24	**2.6E−08**	0.306	**9.63E‐13**	0.224	**8.77E‐07**
Myeloid cDC2	CD1c	0.0061	8.90E‐01	0.012	7.84E‐01	0.047	3.84E‐01
	ITGAM	−0.011	8.10E‐01	0.025	5.69E‐01	0.056	3.05E‐01
	ITGAX	0.11	**1.40E‐02**	0.126	**4.02E‐03**	0.143	**2.90E‐03**
Mo‐DC	CD1a	−0.032	4.70E‐01	−0.006	8.92E‐01	0.008	8.96E‐01
	CD1c	0.0061	8.90E‐01	0.012	7.84E‐01	0.047	3.84E‐01
	ITGAX	0.11	**1.40E‐02**	0.126	**4.02E‐03**	0.143	**2.90E‐03**
Pre‐DC	CLEC4C	0.035	4.20E‐01	0.017	6.96E‐01	0.035	5.90E‐01
	IL3RA	−0.019	6.60E‐01	−0.057	1.94E‐01	−0.037	5.42E‐01
Non‐classical monocyte	CX3CR1	0.046	2.90E‐01	0.043	3.31E‐01	0.075	1.62E‐01
	FCGR3A	0.1	**2.00E‐02**	0.14	**1.41E‐03**	0.144	**2.10E‐03**
	SECISBP2L	0.34	**8.9E−16**	0.308	**7.19E‐13**	0.215	**1.87E‐06**
Langerhans cell	CD1a	−0.032	4.70E‐01	−0.006	8.92E‐01	0.008	8.96E‐01
	CD207	−0.00084	9.80E‐01	−0.001	9.80E‐01	0.009	9.49E‐01
	CDH1	0.19	**1.3E−05**	0.121	**5.65E‐03**	0.095	6.38E‐02

Bold values indicate *p* < 0.05.

### 4.8 Correlation between TTC7B and ferroptosis in HNSCC

**FIGURE 6:**
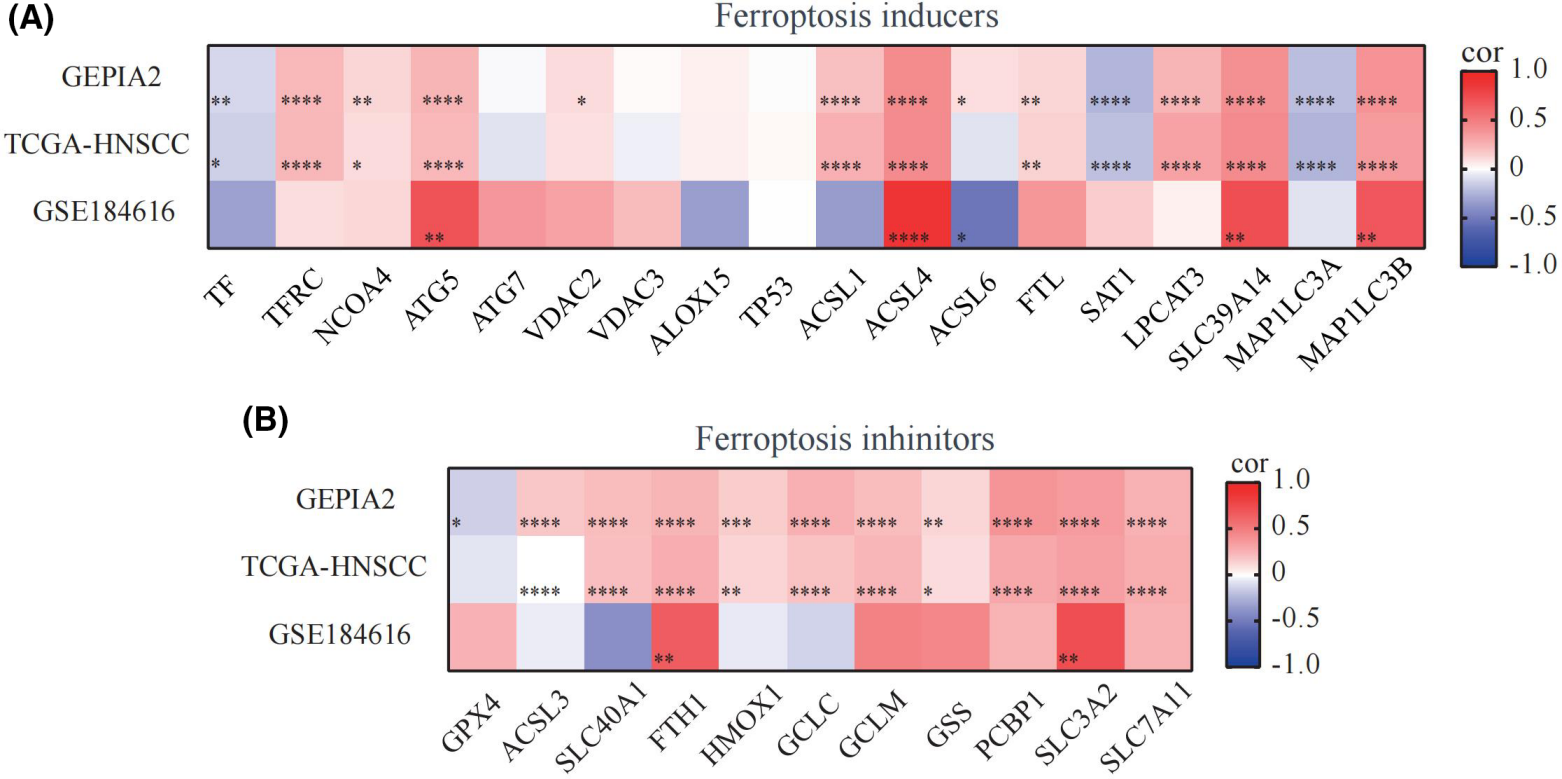
Correlations of TTC7B expression with ferroptosis related genes in Head and neck squamous cell carcinoma (HNSCC). (A) GEPIA2, The Cancer Genome Atlas (TCGA) and GSE184616 datasets analyzed the correlation between the TTC7B and the ferroptosis inducers in HNSCC. (B) GEPIA2, The Cancer Genome Atlas (TCGA) and GSE184616 datasets analyzed the correlation between the TTC7B and the ferroptosis inhibitors in HNSCC. *p < 0.05; **p < 0.01; ***p < 0.001; ****p < 0.0001. NS, not significant.

Ferroptosis was initially identified as a novel form of cell death in 2012.^19^ In contrast to autophagy and apoptosis, ferroptosis is an iron‐dependent and reactive oxygen species (ROS)‐mediated cell death process, characterized primarily by cytological changes, including reduced or absent mitochondrial cristae, a disrupted outer mitochondrial membrane, and a compacted mitochondrial membrane.^20^ Numerous genes and signaling pathways associated with cancer have been found to regulate ferroptosis, and inducing ferroptosis has shown promise for inhibiting tumor progression.^6^ To investigate the correlation between TTC7B expression and ferroptosis, we performed Pearson correlation analysis using the TCGA‐HNSCC, GSE184616, and GEPIA2 datasets. The results revealed a significant correlation between TTC7B expression and both ferroptosis inducer (ATG5, ACSL4, ACSL6, SLC39A14, MAP1LC3B, etc.) and inhibitor (FTH1, SLC3A2, etc.) (Figure 6A, B).

**FIGURE 7:**
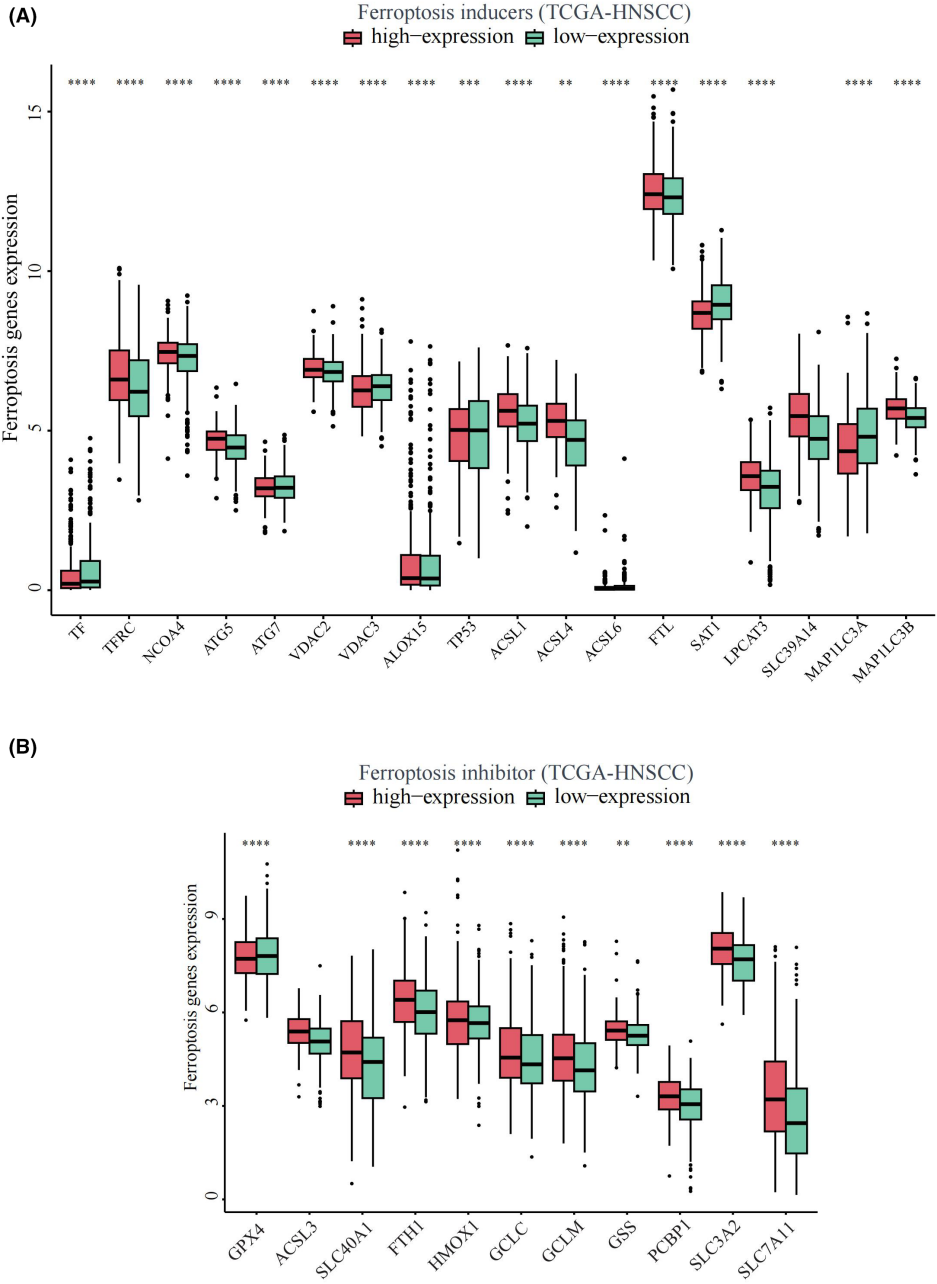
Correlations of TTC7B expression with ferroptosis related genes in Head and neck squamous cell carcinoma (HNSCC). (A) The differential expression of ferroptosis inducers between high and low TTC7B expression groups in HNSCC tumor samples. (B) The differential expression of ferroptosis inhibitors between high and low TTC7B expression groups in HNSCC tumor samples. *p < 0.05; **p < 0.01; ***p < 0.001; ****p < 0.0001. NS, not significant.

Based on the expression levels of TTC7B, we classified samples from TCGA‐HNSCC into high and low expression groups. We then analyzed the differential expression of ferroptosis‐related genes between the two groups in HNSCC (Figure 7A, B). The results from TCGA‐HNSCC showed that the high expression group of TTC7B exhibited increased expressions of ferroptosis inducers, including TFRC, NCOA4, ATG5, VDAC2, ALOX15, TP53, ACSL1, ACSL4, ACSL6, FTL, LPCAT3, and MAP1LC3B (*p* < 0.05). Conversely, the expressions of TF, ATG7, VDAC3, SAT1, and MAP1LC3A in the high expression group of TTC7B were decreased (*p* < 0.05). Additionally, the high expression group of TTC7B showed increased expressions of ferroptosis inhibitors, including SLC40A1, FTH1, HMOX1, GCLC, GCLM, GSS, PCBP1, SLC3A2, and SLC7A11 (*p* < 0.05), while GPX4 expression in the high expression group of TTC7B was decreased (*p* < 0.05). These findings indicate a strong correlation between TTC7B expression and both ferroptosis inducers and inhibitors in HNSCC. The potential role of TTC7B in regulating ferroptosis may affect the progression and prognosis of HNSCC.

## 5 DISCUSSION

Despite significant advancements in multidisciplinary approaches for the treatment of HNSCC, managing this disease remains a formidable challenge, as many patients face poor prognosis and encounter significant physical and financial burdens, especially those in advanced stages of the disease.^21^ Therefore, identifying novel prognostic‐related biomarkers and elucidating their functions are crucial for improving clinical treatment and patient outcomes. TTC7B, a protein‐coding gene involved in the phosphatidylinositol phosphate biosynthetic process and protein localization to the plasma membrane, has recently been recognized as a potential prognostic biomarker for glioma.^4^ While previous investigations did not report the involvement of TTC7B gene in HNSCC, we aimed to evaluate its role in HNSCC.

Through the analysis of TIMER2.0 online database, we found TTC7B was low expressed in 12 types of cancer and overexpressed in 3 tumors. Based on the analysis of TCGA‐HNSCC and GEO datasets, the expression level of TTC7B in HNSCC tissues was significantly higher than that in normal tissues. qPCR and IHC experiments confirmed the upregulated expression of TTC7B in both HNSCC cell lines and tissues. We systematically stratified HNSCC based on their precise anatomical sites, and intriguingly, we noted that heightened expression of TTC7B in HNSCC originating in the pharynx was not significantly linked to a poor prognosis. This observation implies the existence of some degree of heterogeneity within HNSCC depending on the anatomical site. Additionally, our analysis unveiled a noteworthy difference in TTC7B expression between HPV‐negative and HPV‐positive HNSCC patients, with TTC7B exhibiting significantly higher levels in the former group. This observation suggests that TTC7B may play a more prominent role in the context of HPV‐negative patients. We also used ROC curve analysis to assess the predictive capacity of TTC7B expression for HNSCC and found that it had the potential to discriminate between tumor and normal samples. Previous research demonstrated that low TTC7B expression was linked to an unfavorable OS in glioma.^2^ However, our findings based on GEPIA2 and TCGA HNSCC datasets showed the opposite result in HNSCC, suggesting that modulating the expression level of TTC7B may have implications for improving the prognosis of patients with HNSCC. The differing results for TTC7B in glioma and HNSCC may be attributed to the distinct origins of these two tumor types, as well as their varying mechanisms of development. In conclusion, TTC7B has the potential to serve as a valuable diagnostic and prognostic marker for HNSCC.

The functional analysis of TTC7B in tumors has been relatively limited. Therefore, in this study, we employed Pearson correlation analysis to identify co‐expressed genes of TTC7B in HNSCC based on the TCGA‐HNSCC and GSE184616 datasets. The GO and KEGG function enrichment analysis of genes with the top 500 R‐values and *p* < 0.05 demonstrated that the expression of TTC7B in HNSCC was associated with cell migration, FAs, cell shape, etc. Given the significant role of FAs‐related genes in tumor cell invasion and migration, we further investigated the relationship between TTC7B expression and FAs‐related pathways in HNSCC using GSVA.^16^ Results showed a positive linkage between TTC7B and FAs in HNSCC, especially the PINCH‐ILK‐PARVIN complex and FAs disassembly. FAs‐related genes have been known to be elevated in various cancer and associated with cell migration in cancer cells. Additionally, FAs disassembly has been reported to enhance the metastatic ability of cancer cells.^22^, ^23^ While the precise role of FAs in HNSCC remains to be fully elucidated, our findings suggest that TTC7B may contribute to HNSCC invasion and metastasis through FAs disassembly, ultimately leading to a poorer prognosis for HNSCC patients.

Immune infiltration of tumor cells is link to lymph node metastasis and the prognosis of HNSCC.^24^ To explore this further, we conducted CIBERSORT analysis to determine the proportions of 22 tumor immune cell types in HNSCC. The results revealed a significant correlation between the expression level of TTC7B and tumor immune cell infiltration, indicating a potential involvement of TTC7B in modulating the immune microenvironment in HNSCC. Additionally, TIMER2.0 database analysis further showed that the expression level of TTC7B in HNSCC was positively correlated with myeloid dendritic cell and macrophages, and negatively correlated with CD4^+^ T cell. These findings highlight the important role of TTC7B in modulating the tumor immune microenvironment in HNSCC. Next, we explored the association between TTC7B expression and marker genes of various immune cells. And as we can see from Table 2, the expression level of TTC7B had the strongest correlation with the expression of macrophage‐related genes. Macrophages, acting as double‐edged swords in cancer, can both eliminate tumor cells and contribute to cancer progression and metastasis through various mechanisms, including promoting cancer cell survival and proliferation, angiogenesis, and suppressing innate and adaptive immune responses.^25^, ^26^, ^27^ Recent studies have also demonstrated the promotion of cancer progression by macrophages in HNSCC.^28^, ^29^ Therefore, we speculate that overexpression of TTC7B might promote cancer progression by increasing macrophages levels in HNSCC. Nevertheless, to gain a better understanding of the interaction between TTC7B and macrophages in vivo, more experiments are required in the future.

Targeting ferroptosis has garnered great interest in the field of cancer therapy, as inducing ferroptosis might present a potential avenue for treating refractory tumors.^30^ Several tumor suppressors, including P53,^31^ BAP1,^32^ and fumarase,^33^ have been identified for their ability to sensitize cells to ferroptosis. In this context, we explored the relationship between TTC7B expression and ferroptosis‐related genes, and intriguingly, we found a positive correlation between TTC7B expression and both ferroptosis inducers and inhibitors. These significant findings suggest that TTC7B's role in promoting cancer in HNSCC may be linked to its involvement in the regulation of ferroptosis. As ferroptosis modulation presents a potential avenue for cancer treatment, understanding the mechanisms by which TTC7B influences this process could open up new possibilities for therapeutic interventions in HNSCC.

However, this study does have some limitations. Most of the data used in our analysis were sourced from publicly available databases, and there is a dearth of experimentally validated data. Furthermore, there is room for more comprehensive investigations into functional enrichment, immune infiltration, and ferroptosis‐related aspects. The precise mechanisms underlying TTC7B's interactions with FAs, macrophages, and ferroptosis‐related genes have not been fully elucidated. In future research, we plan to conduct more in‐depth inquiries into how TTC7B specifically impacts the prognosis of HNSCC patients, building upon the foundations established in this study.

## 6 CONCLUSION

Our study provides compelling evidence supporting the overexpression of TTC7B in HNSCC, and importantly, we observed a strong association between high TTC7B expression and a poor prognosis in HNSCC patients. Notably, we identified a significant correlation between TTC7B and FAs, particularly FAs disassembly, which has been linked to tumor invasion and metastasis. Furthermore, our study unveiled a close relationship between TTC7B expression and the extent of immune cell infiltration, potentially contributing to tumor migration and invasion by facilitating macrophage infiltration. The intriguing link between TTC7B and ferroptosis‐related genes sheds light on why elevated TTC7B expression is indicative of a poor prognosis in HNSCC. Collectively, our findings highlight the potential of TTC7B as a promising biomarker for the diagnosis, treatment, and prognosis of HNSCC.

## AUTHOR CONTRIBUTIONS

**Rong He:** Conceptualization (equal); data curation (lead); methodology (lead); writing – original draft (lead). **Xun Zhang:** Methodology (equal); supervision (equal); writing – review and editing (equal). **Yongzhi Wu:** Methodology (equal); supervision (equal); writing – review and editing (equal). **Zhijie Weng:** Methodology (equal); supervision (equal); writing – review and editing (equal). **Longjiang Li:** Conceptualization (equal); funding acquisition (lead); writing – review and editing (equal).

## FUNDING INFORMATION

This study was supported by the National Natural Science Foundation of China (No. 82141130).

## CONFLICT OF INTEREST STATEMENT

No potential conflict of interest was reported by the authors.

## ETHICS STATEMENT

The protocol of this study had been approved by the Ethics Committee of West China Hospital of Stomatology, Sichuan University, Chengdu, China (No. WCHSIRB‐D‐2023‐146). Informed consent was obtained before collecting human samples.

## Supporting information


Figure S1.

Table S1.

## Data Availability

The datasets used or analyzed during the current study are available from the corresponding author upon reasonable request.
