# Supplementary figures and images for: TTC7B is a new prognostic biomarker in head and neck squamous cell carcinoma linked to immune infiltration and ferroptosis
Source: Cancer Med. 2023 Nov 22;12(24):22354–69. doi: 10.1002/cam4.6715 (PMC10757123; doi:10.1002/cam4.6715)

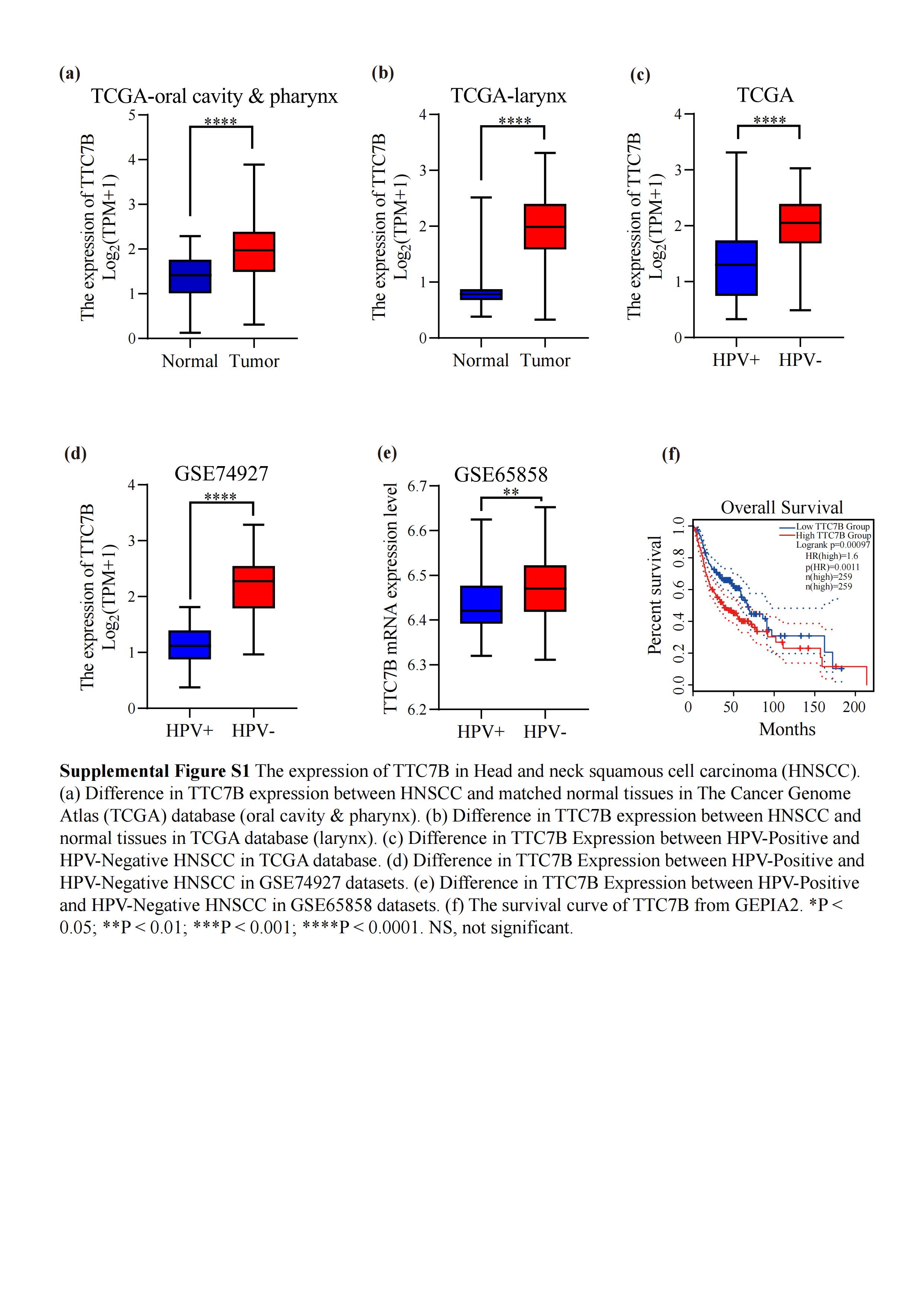

Supplement: Supplementary file 1 — Figure S1. [file CAM4-12-22354-s002.jpg]
