# Supplementary material for: TTC7B is a new prognostic biomarker in head and neck squamous cell carcinoma linked to immune infiltration and ferroptosis
Source: Cancer Med. 2023 Nov 22;12(24):22354–69. doi: 10.1002/cam4.6715 (PMC10757123; doi:10.1002/cam4.6715)
Supplement: Supplementary file 2 — Table S1. [file CAM4-12-22354-s001.docx]

**Supplemental Table S1** Survival analysis of TTC7B in different sites of HNSCC

| Datasets | HR | 95% CI | P |
| --- | --- | --- | --- |
| TCGA-HNSCC (oral cavity & pharynx) | 1.452 | 1.150-1.834 | 0.002 |
| TCGA-HNSCC (larynx) | 1.315 | 0.881-1.963 | 0.181 |
| GSE41634 (oral cavity) | 1.567 | 1.061-2.314 | 0.024 |
